# Osteoporosis treatment: the battle to be won

**DOI:** 10.20945/2359-3997000000276

**Published:** 2020-08-06

**Authors:** Joao Lindolfo C Borges

**Affiliations:** 1 Academia Brasileira de Medicina da Reabilitação Brasil Membro da Academia Brasileira de Medicina da Reabilitação. Membro da Academia Brasileira de Medicina Militar. Fellow do Colégio Americano de Endocrinologia. Presidente da Sociedade Brasileira de Endocrinologia e Metabologia (SBEM-DF), Brasília, DF, Brasil; Academia Brasileira de Medicina Militar Brasil; Colégio Americano de Endocrinologia Brasil; Sociedade Brasileira de Endocrinologia e Metabologia Brasília DF Brasil

**Figure 1:**
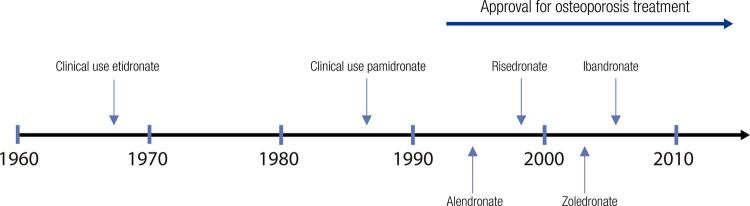
Brief history of biphosphonates. Modified from Bone. 2011;49(1):2-19.

Bisphosphonates (BPs) are the pioneer and gold standard drugs for osteoporosis treatment. The development of BPs started decades ago. There are four bisphosphonates in the osteoporosis market: alendronate, risedronate, ibandronate, and zolendronate. Pamidronate, an important BP, is used for the treatment of osteogenesis imperfecta ( 1 ). Figure 1 shows the evolution of BPs discovery and approval for osteoporosis treatment.

Healthy bone growth and remodeling entail a tightly coupled process of bone resorption and new bone formation. Bone loss leading to osteoporosis occurs when bone resorption is more significant than bone formation; BPs decrease bone resorption, thereby slowing bone loss with a minor decline in bone-forming action. The pharmacology of BPs is complex. BPs are incorporated into newly formed bone and can persist there for decades, through multiple cycles of bone resorption and deposition. Patients keep on being exposed to the pharmacologic impacts of BPs action long after they quit taking the medication ( 2 ).

Millions of Americans are passing up an opportunity to abstain from fractures from low bone mass since they are scared of extremely uncommon reactions from drugs that can help them ( 3 ).

Claims over the uncommon symptoms brought about huge jury grants and drew boundless consideration. Furthermore, after reports of these issues started to surface, the Food and Drug Administration requested that the drugs’ labels include a warning about the association.

Adequacy of BPs treatment for osteoporosis is an essential concern for the Food and Drug Administration (FDA). Considering postmarketing reports of uncommon however genuine adverse events related with BPs, for example, atypical femur fractures, osteonecrosis of the jaw, and esophageal malignancy.

When all of the evidence is considered, the anti-fracture benefit provided by the amino-BPs far outweighs the potential risks of therapy in most patients at high risk of fracture.

Depending on the seriousness of osteoporosis, somewhere in the range of 9 and 60 patients should be treated for a long time to forestall one vertebral fracture; somewhere in the range of 20 and 68 patients should be treated for a long time to avoid one nonvertebral fracture. In any event, limiting the expanded danger of fracture with propelling age, the number needed to treat for 8 years would be somewhere in the range of 3 to 23 women to avoid a vertebral fracture, and somewhere in the range of 7 to 26 women for nonvertebral fracture. Considering the scope of hazard gauges for osteonecrosis of the jaw, one case would happen for each 1,000 to 100,000 patients treated. Using the data from California,47 one atypical femoral fracture would occur in 1,282 patients treated for 8 years. Based on the Swedish data, 8 years of therapy would result in one atypical femoral fracture for every 149 patients treated (8.4 cases/10,000 patient-years) ( 4 ).

Clearly, given the potential for combined hazards, alert ought to be practiced in exchanging among BPs and other potent antiresorptive drugs. Further examination concerning the advantages and dangers of long-term treatment, just as surveillance of fracture risk after suspension of BPs treatment, will be essential for deciding the best routine of therapy for individual patients with osteoporosis.

In this issue, Bandeira and cols. ( 5 ) nicely reviewed the efficacy/safety of long term BPs use. The authors conclude that until a risk calculator for predicting the risk of atypical fractures becomes available in clinical practice. It is up to the physician to consider continuing or discontinuing BPs use after the critical 3-5 year period of treatment with zoledronic acid or alendronate. The treatment of osteoporosis is a long battle to be won.
